# Aberrant brain grey matter volume patterns differ among Chinese Han drug-naïve depression patients with acute and chronic stress

**DOI:** 10.18632/oncotarget.20954

**Published:** 2017-09-16

**Authors:** Ping Guo, Shikai Wang, Ce Chen, Hongjun Tian, Jie Li, Weifang Zheng, Mincai Qian

**Affiliations:** ^1^ Department of Psychological Medicine, Huzhou Third People's Hospital, Huzhou, China; ^2^ Department of Psychological Medicine, Tianjin Anding Hospital, Tianjin, China; ^3^ Department of Psychological Medicine, Wenzhou Seventh People's Hospital, Wenzhou, China

**Keywords:** depression, stress, grey matter volume, aberrant pattern

## Abstract

Chronic or acute stress can induce structural changes and brain alterations associated with the neural mechanisms of depression. Aimed to investigate the GMV alterations in the drug-naïve depression patients with chronic and acute stress experience,we enrolled fifty depression patients with acute stress experience, fifty five depression patients with chronic stress experience and forty seven healthy controls(HC) to participant in the present study. We used voxel-based morphometry to analyze the brain grey matter volume (GMV) alterations. Compared with the HC, the patients with acute stress and those with chronic stress exhibited a distinct GMV impairment pattern. Widespread, decreased GMV was detected in most of the cerebral cortex in all the depression patients. Importantly, the greatest finding in our study is that the decreased GMV in the depression patients with chronic stress was more widespread than that in the patients with acute stress. All brain regions with decreased GMV participated in the regulation of emotions, memory, and executive function processing, which is consistent with previous findings. There was no significant difference between the major depression disorder patients with acute stressful life events and those with chronic stressful life events, and this finding largely weakens the support of our current conclusion. Thus, we cannot confirm this postulation. However, our findings probably indicate that GMV may be more sensitive to major depression disorder patients when compared to healthy controls, it did not sensitive when in the comparison of patient's group. Overall, our findings provide important information for the use of appropriate treatment methods to address acute stress and alleviate chronic stress in patients with depression, and such treatments can delay the deterioration of the affected brain regions and improve remission rates. More importantly, all the inexplicable findings in the present study encourage us to conduct a follow-up study to describe the developmental trajectory of the pathological brain features of depression patients and explore therapeutic targets for future personalized treatment.

## INTRODUCTION

Before the onset of depression, most patients experience a certain degree of chronic mild stress [1]. Chronic stress can influence structural abnormalities in the brain in animal models of depression [2], and stressful life events can induce a decrease in GMV in the anterior cingulate, hippocampus, parahippocampal gyrus, bilateral caudate nucleus and thalamus [1, 3, 4]. These abovementioned brain regions play key roles in the pathological mechanisms of depression [1–5]. Moreover, to some extent, the pattern of structural abnormalities found in the brains of depressed patients are similar to the aberrant patterns in patients diagnosed with post-traumatic stress disorder, which is induced by extreme, acute stress [3–7].

Some previous brain imaging studies based on magnetic resonance imaging (MRI) found structural and functional aberrations between depression patients and healthy controls [8–13]. For example, the prefrontal area and limbic system demonstrate significant structural deficits, and the anterior cingulate cortex; ventromedial prefrontal cortex; dorsolateral prefrontal cortex; lateral orbital prefrontal cortex; and some subcortical regions, such as the amygdala, hippocampus and ventral striatum, are also affected in depression patients [14, 15]. In addition, except for the GMV alterations observed in depression patients, regional blood flow disturbances are also observed in brain regions related to depression [16–19]. Moreover, depression patients have abnormal levels of white matter (WM) [20–22]. Almost all the aforementioned reports converge to suggest that the brain areas involved in the circuit that participates in the processing of emotion regulation, memory and executive function regulation are all affected in depression, as demonstrated by multiple indices, such as GMV, WM, and cerebral blood flow (CBF) [1, 23].

Collectively, the previous findings suggest that chronic or acute stress can induce structural changes in the brain or CBF alterations associated with the neural mechanisms of depression. However, few studies have compared the differences in brain structural alterations between depression patients experiencing chronic stress and those experiencing acute stress. Based on previous studies, in the present study, we compared GMV differences among drug-naïve depression patients with chronic and acute stress factors before the onset of depression. We postulated that the altered patterns of GMV are distinct between these two groups of depression patients.

## RESULTS

### Comparison of the GMV alterations between depression patients with acute stress and healthy controls

**Figure 1:**
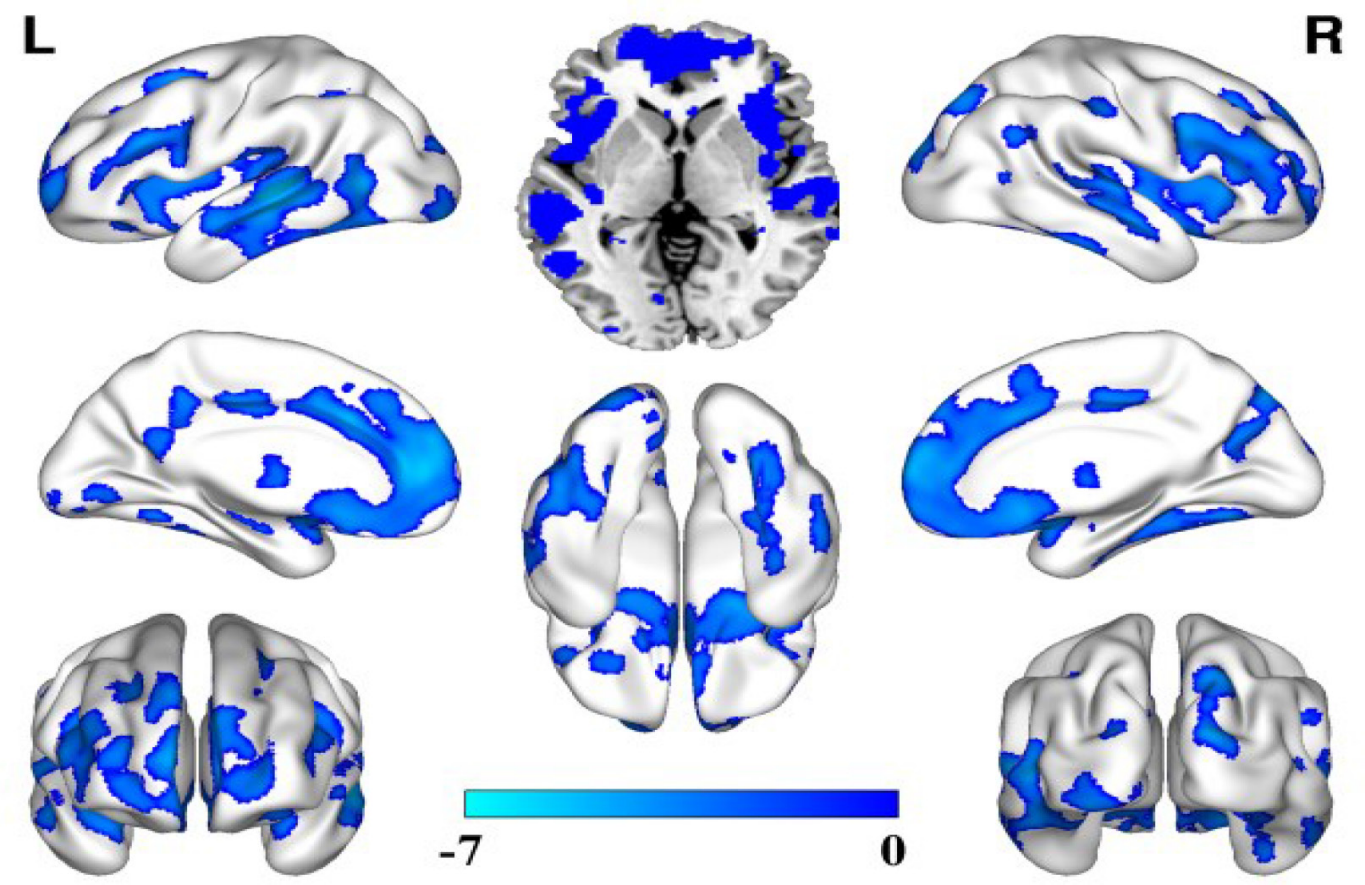
GMV decreased in the depression patients with acute stress compared with that in the healthy controls (false discovery rate (FDR) correction, *P* < 0.05, cluster size = 100)

Compared with the healthy controls, the depression patients with acute stress showed significantly decreased GMV in brain regions throughout the bilateral frontal lobes, such as the bilateral temporal lobes, prefrontal lobes, occipital lobe, and parietal lobes (Figure 1).

### Comparison of the GMV alterations between depression patients with chronic stress and healthy controls

**Figure 2:**
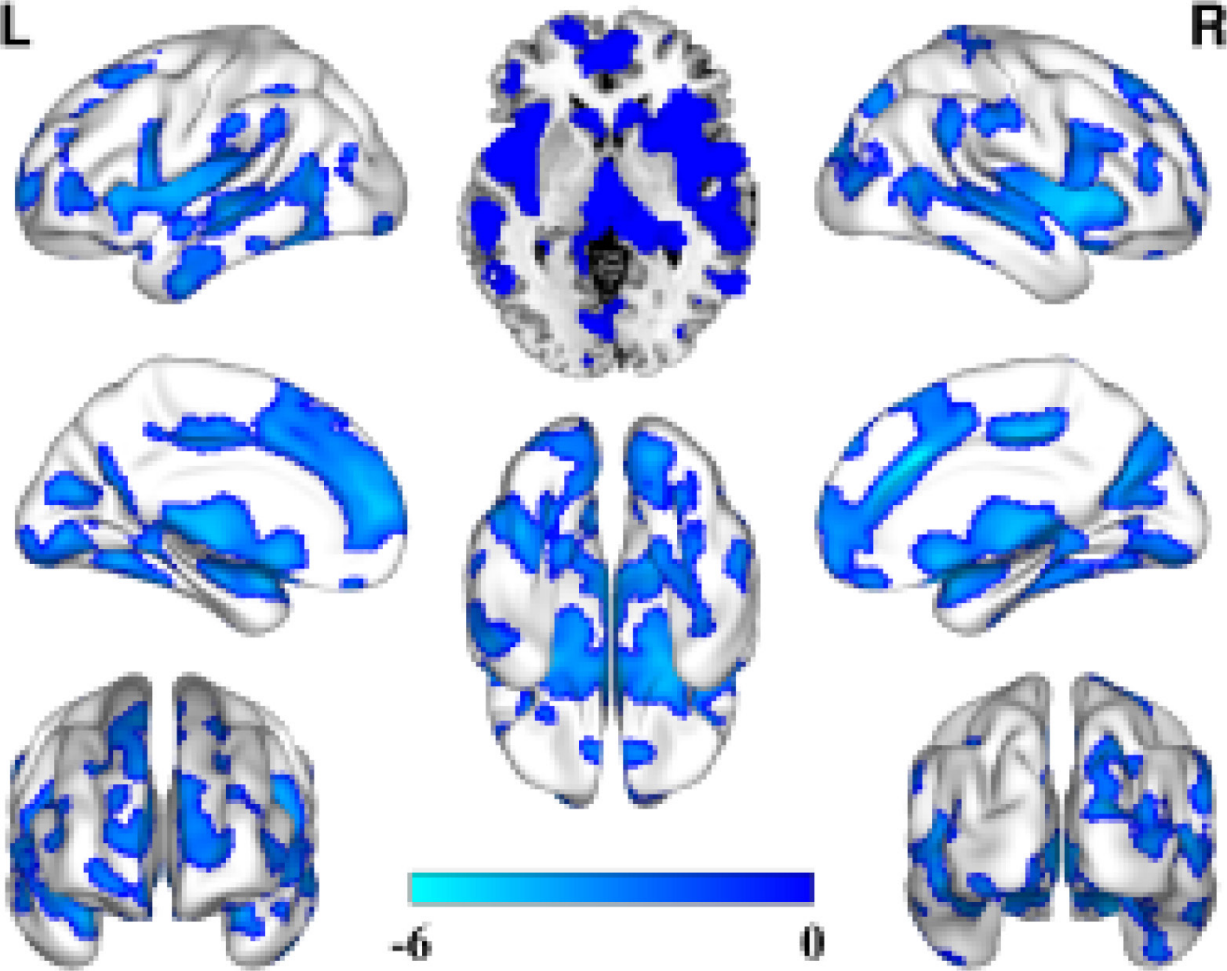
GMV decreased in the depression patients with chronic stress compared with that in the healthy controls (FDR correction, *P* < 0.05, cluster size = 100)

Compared with the healthy controls, unlike the depression patients with acute stress, those with chronic stress showed significantly decreased GMV in brain regions in the bilateral prefrontal lobe, including the temporal lobe, parietal lobes and occipital lobe. More importantly, the bilateral basal ganglia and thalamus also demonstrated significantly decreased patterns of GMV (Figure 2).

## DISCUSSION

In the present study, we first compared GMV impairment in drug-naïve depression patients with acute stress and those with chronic stress. Compared with the healthy controls, the patients with acute stress and those with chronic stress exhibited distinct GMV impairment patterns. Most of the cerebral cortex showed widespread, decreased GMV. All the brain regions with decreased GMV participated in the regulation of emotions, memory, and executive function processing [1, 23], and this result is consistent with previous findings. However, the greatest finding in our study is that the decreased GMV in the depression patients with chronic stress is more widespread than that in the patients with acute stress, demonstrating that long-term chronic stress affects brain structures more than does acute stress and supporting the hypothesis that anxiety damages the brain [24]. Therefore, we should adopt optimal treatments to control the influence of acute stress when treating depression patients and adopt multiple methods, such as social support and psychological therapy, to prevent the perpetuation of acute stress and to protect the brain from further deterioration [25–27]. Additionally, our findings support the use of multiple treatment methods to alleviate the influence of chronic stress in depression patients, which can also delay the deterioration of affected brain regions [25, 28].

A strength of this study is that we enrolled drug-naïve depression patients, thereby lessening the influence of previous therapy and allowing the results to objectively reflect the pathological characteristics of depression patients. The overall GMV decreased in the two patient groups in the prefrontal lobe and temporal, parietal and occipital lobe areas. Specific GMV alterations in the patients with chronic stress were observed in the bilateral basal ganglia and thalamus. Functional or structural alterations in the frontal-limbic network influence the regulation of affect and memory processing and impair executive function to some extent[1, 23, 29]. Our present findings and previous findings support the GMV impairment hypothesis of depression.

Some inexplicable findings were observed in our present study. There was no significant difference between the depression patients with acute stress and those with chronic stress after FDR correction, but compared with the patients with acute stress, the patients with chronic stress showed decreased GMV in the right insular lobe, right inferior frontal gyrus, right inferior temporal gyrus, and left anterior cingulate cortex without FDR correction. To the best of our knowledge, we postulated that there are two potential reasons maybe explain this phenomenon. First, the GMV impairment maybe the common brain feature of MDD, it is unrelated to the stressful life events experience or not. Second, we missed the best time point for brain imaging scanning in the MDD patients with acute stressful life events experience. The time delay maybe caused the specific GMV alterations disappeared in the MDD patients with acute stress. Frankly, the above two postulated reasons are untenable. This unique phenomenon cannot be explained in the current study and warrants further investigation.

Since the abovementioned quandary still needs to be resolved, we need to conduct a high-quality, long-term, follow-up study to explore the dynamic structural and functional aberrations in different categories of depression patients from the first episode of depressive symptoms to determine the appropriate treatment and remission parameters. Then, we could describe the pathological brain characteristics in the developmental trajectory of depressive disorder and explore therapeutic targets for personalized treatment.

## MATERIALS AND METHODS

### Subjects

All patients were diagnosed by a psychiatrist using the Structured Clinical Interview (SCID) according to the criteria for major depressive disorder in the Fourth Edition of the Diagnostic and Statistical Manual of Mental Disorders, Text Revision (DSM-IV, TR Version). The criteria for acute and chronic stress were also used by a psychiatrist according to relative reports and medical reports. An acute stress experience was defined as the experience of negative stressful events in the last six months before depression onset that currently remain active. Chronic stress was defined as the experience of negative stressful events in the last 24 months before depression onset that currently remain active. Both acute stress and chronic stress patients all attribute depression onset to stressful life events. Simultaneously, we adopted the life stressful events scale (LSE) to qualitatively and qualitatively assess the negative effects of acute and chronic stressful life events on the patients [30]. All the healthy controls were excluded by a psychiatrist using the SCID-I/Non-patient (NP Version). The 17-item version Hamilton Rating Scale for Depression (HAMD) [31] was used to evaluate the severity of depressive symptoms. A total of 105 patients were enrolled in the present study from January 2012 to December 2016. The mean age of the patients with acute stress was 43.6 ± 4.5 years, and the mean illness duration was 7.8 ± 2.8 (months). The mean age of the patients with chronic stress was 44.5 ± 6.1 years, and the mean illness duration was 36.2 ± 5.3 (months). Forty-seven healthy controls were recruited from the hospital staff of Huzhou 3rd Hospital (Huzhou, Zhejiang) and Wenzhou Seventh People's Hospital (Wenzhou, Zhejiang), with a mean age of 43.7 ± 3.6 years. The psychiatrists also ruled out a diagnosis of potential mental disorder, and the healthy controls were enrolled only if they did not have a positive family history of mental disorder. The exclusion criteria were as follows: a history of unconsciousness for ≥ 5 min, neurological disease, other severe mental disorders, drug abuse, serious physical disease, pregnancy or lactation, participation in any other research study, and endocrine disease or any other contraindication for MRI scanning. The Ethics Committee of Hu Zhou 3rd Hospital and Wenzhou Seventh people's Hospital all approved the current study. All participants fully understood the current study purposes and provided written informed consent. All the patients experienced a two-week inpatient admission to washout any previous therapeutic effects. Table 1 shows the demographic information of all the participants. The illness duration and severity of the depressive symptoms of the patients with chronic stress are all significantly different from those of the healthy controls.

**Table 1:** Demographic and clinical characteristics of the participants

	DD+AS (*n* = 50)	DD+CS (*n* = 55)	HC (*n* = 47)	*P*-value
Age (yrs)	43.6 ± 4.5	44.5 ± 6.1	43.7 ± 3.6	> 0.05
Sex (M/F)	22/28	25/30	27/20	> 0.05
Illness duration, months	7.8 ± 2.8 (months)	36.2 ± 5.3 (months)		< 0.05
HRSD score	33.5±7.2	47.8 ± 4.6		< 0.05

DD+AS: Drug-naïve depression patients with acute stress; DD+CS: Drug-naïve depression patients with chronic stress; HC: healthy controls; HRSD: 17-item version of the Hamilton Rating Scale for Depression.

### Methods

#### MRI image acquisition

A Philips Achieva 3.0T MRI system (Philips Medical Systems Nederland B.V., the Netherlands) was used to perform the scans. All the subjects were in a comfortable position and were provided special earplugs to reduce the impact of scanner noise. A soft foam pad was placed around each subject's head to reduce head movement. The MRI canning parameters were as follows. Three-dimensional T1-weighted magnetic resonance images were acquired in the sagittal plane using a 3D spoiled gradient recalled acquisition in steady state (SPGR) sequence (TR = 8.3 ms; TE = 3.2 ms; flip angle = 11°; TI = 500 ms; number of excitations (NEX) = 1; array coil spatial sensitivity encoding (ASSET) = 1.5; and frequency direction: S/I). A total of 180 contiguous 1-mm slices were acquired with a 256 × 256 matrix, with an in-plane resolution of 1 mm × 1 mm resulting in isotropic voxels.

#### Magnetic resonance imaging data analysis

All images were corrected for image distortion due to gradient non-linearity using “GradWarp” [32] and for intensity inhomogeneity using “N3” [32]. Image processing for voxel-based morphometry (VBM) [33, 34], a fully automatic technique used for the computational analysis of differences in regional brain volumes throughout the entire brain, was performed with Statistical Parametric Mapping 8 (SPM8; Institute of Neurology, London, UK). The 3D-FSPGR images in native space were spatially normalized and segmented into grey matter (GM), WM, and cerebrospinal fluid (CSF) images and intensity modulated using the Diffeomorphic Anatomical Registration Through Exponential Lie Algebra (DARTEL) toolbox in a high-dimensional normalization protocol. The DARTEL toolbox has been proposed by Ashburner [35] as an alternative method for normalization in SPM. In an intensity-modulation step, the voxel values of the segmented images were multiplied by the measure of the warped and unwarped structures derived from the nonlinear step of the spatial normalization. This step converted the relative regional GM density into the absolute GM density expressed as the amount of GM per unit volume of brain tissue before spatial normalization. The resulting modulated GM and WM images were smoothed with an 8-mm Gaussian kernel.

### Statistical analysis

SPSS 19.0 statistical analysis software (SPSS, Inc., Chicago, IL, USA) was used for the statistical analyses. Continuous variables are presented as the mean±standard deviation (SD). Continuous and categorical variables were compared between the groups using independent samples *t*-tests and Chi-square analysis, respectively. A *P*-value of < 0.05 was considered statistically significant. Age, gender, illness duration and depression severity were used as covariates in the statistical analyses to control for the influence of differences in illness duration. Total GM volume was also compared between the groups using unpaired two-tailed *t*-test.

## CONCLUSIONS

In the present study, we used voxel-based morphometry (VBM) to investigate the differences in brain grey matter volume (GMV) changes between first-episode, drug-naïve, depression patients with and without chronic and acute stress. Compared with the healthy controls, the patients with acute stress and those with chronic stress exhibited a distinct GMV impairment pattern. Widespread, decreased GMV was detected in most of the cerebral cortex in all the depression patients. Importantly, the greatest finding in our study is that the decreased GMV in the depression patients with chronic stress is more widespread than that in the patients with acute stress. All brain regions with decreased GMV participated in the regulation of emotions, memory, and executive function processing, and this result is consistent with previous findings. There was no significant difference between the major depression disorder (MDD) patients with acute stressful life events and those with chronic stressful life events, and this finding largely weakens the support of our current conclusion. Thus, we cannot confirm this postulation. However, our findings probably indicate that GMV may be more sensitive to MDD patients when compared to healthy controls, it did not sensitive when in the comparison of patient's group. Overall, our findings provide important information for the use of appropriate treatment methods to address acute stress and alleviate chronic stress in patients with depression, and such treatments can delay the deterioration of the affected brain regions and improve remission rates. More importantly, all the inexplicable findings in the present study encourage us to conduct a follow-up study to describe the developmental trajectory of the pathological brain features of depression patients and explore therapeutic targets for future personalized treatment.
